# Structural Validation of a French Food Frequency Questionnaire of 94 Items

**DOI:** 10.3389/fnut.2017.00062

**Published:** 2017-12-20

**Authors:** Rozenn Gazan, Florent Vieux, Nicole Darmon, Matthieu Maillot

**Affiliations:** ^1^MS-Nutrition, Marseille, France; ^2^UMR NORT (Unité Mixte de Recherche – Nutrition, Obesity and Risk of Thrombosis), Aix-Marseille Université, INSERM, INRA 1260, Marseille, France; ^3^UMR MOISA (Markets, Organizations, Institutions and Stakeholders Strategies), INRA 1110, Université de Montpellier, France

**Keywords:** food intakes, nutritional intakes, portion sizes, measurement error, simulation

## Abstract

**Background:**

Food frequency questionnaires (FFQs) are used to estimate the usual food and nutrient intakes over a period of time. Such estimates can suffer from measurement errors, either due to bias induced by respondent’s answers or to errors induced by the structure of the questionnaire (e.g., using a limited number of food items and an aggregated food database with average portion sizes). The “structural validation” presented in this study aims to isolate and quantify the impact of the inherent structure of a FFQ on the estimation of food and nutrient intakes, independently of respondent’s perception of the questionnaire.

**Methods:**

A semi-quantitative FFQ (*n* = 94 items, including 50 items with questions on portion sizes) and an associated aggregated food composition database (named the item-composition database) were developed, based on the self-reported weekly dietary records of 1918 adults (18–79 years-old) in the French Individual and National Dietary Survey 2 (INCA2), and the French CIQUAL 2013 food-composition database of all the foods (*n* = 1342 foods) declared as consumed in the population. Reference intakes of foods (“REF_FOOD”) and nutrients (“REF_NUT”) were calculated for each adult using the food-composition database and the amounts of foods self-reported in his/her dietary record. Then, answers to the FFQ were simulated for each adult based on his/her self-reported dietary record. “FFQ_FOOD” and “FFQ_NUT” intakes were estimated using the simulated answers and the item-composition database. Measurement errors (in %), spearman correlations and cross-classification were used to compare “REF_FOOD” with “FFQ_FOOD” and “REF_NUT” with “FFQ_NUT”.

**Results:**

Compared to “REF_NUT,” “FFQ_NUT” total quantity and total energy intake were underestimated on average by 198 g/day and 666 kJ/day, respectively. “FFQ_FOOD” intakes were well estimated for starches, underestimated for most of the subgroups, and overestimated for some subgroups, in particular vegetables. Underestimation were mainly due to the use of portion sizes, leading to an underestimation of most of nutrients, except free sugars which were overestimated.

**Conclusion:**

The “structural validation” by simulating answers to a FFQ based on a reference dietary survey is innovative and pragmatic and allows quantifying the error induced by the simplification of the method of collection.

## Introduction

In nutritional intervention studies, reliable dietary data are essential to avoid misleading conclusions. Food intakes are generally assessed using short-term instruments (i.e., 24 h-recall, dietary records) or long-term instruments such as food frequency questionnaires (FFQs) (1). A FFQ is a retrospective instrument where the respondent has to report the frequency of consumption on a predefined list of food items (an item could be an individual food or an aggregation of same kind of foods), during a more or less long period of time (1 month to 1 year) (1). A FFQ may be qualitative when it does not collect information on the quantity consumed, semi-quantitative if it contains standard portions sizes or quantitative when it includes questions about portion sizes consumed. FFQs require a short time to complete, are less burdensome for respondents, and are less expensive to setup than short-term instruments (2, 3). FFQs were shown to be valuable tools to estimate dietary changes in nutritional intervention studies (4) and they remain one of the most common dietary measurement tools in dietary intervention studies to capture the usual food and nutrient intakes over a period of time (5–7).

However, the accuracy of the nutritional intakes estimated using FFQs has been fully in debate (8–12). Dietary estimation relies on a difficult cognitive task for the respondent, to remember the frequency and, when necessary, portion sizes of foods consumed in the past. Moreover, food and nutrient estimates could be biased by errors inherent to the FFQ (3, 13, 14). Nutrient intakes from a FFQ are estimated by multiplying the frequency of consumption of each food item by its respective portion size and nutritional content. Food consumption is collected for a closed list of items and, therefore, cannot fully capture in detail an individual’s diet. Quantification of the food consumed does not account for the variability of portion sizes across eating occasions or between specific foods related to the same item. Each item composing the FFQ is an aggregation of different foods with different nutrient contents. The nutrient content of an item is, in general, a weighted mean nutritional composition of all foods represented in the item, taking into account the amount consumed, to reflect the foods actually eaten in the population of interest (15). Therefore, the error in the estimation of food and nutrient intakes could either be due to imprecision in the nutritional content of food items or to imprecision in the portion sizes used. Because of known systematic errors in a FFQ and because the “true” intake is unknown, the validation of a FFQ is necessary. It is usually done by comparing the estimation of food and nutrient intakes against “a gold standard,” the latter often being a short-term open-ended instrument (16). Such comparison allows identifying the sources and magnitudes of the measurement error but cannot distinguish between the error due to the inherent structure of the FFQ or due to the differential in the respondent perception of the two instruments. This work describes a new method called “structural validation,” which allows isolating and quantifying the impact of the inherent structure of a given questionnaire on the estimation of food and nutrient intakes, independently of respondent’s perception of the questionnaire.

## Materials and Methods

### Survey Design and Estimation of “REF_FOOD” and “REF_NUT” Intakes

Food intakes were derived from the French Individual and National Dietary Survey 2 (INCA2) conducted in 2006–2007 by the French Agency for Food, Environmental and Occupational Health Safety, performed on nationally representative samples of children (3–17 years) and adults (18–79 years). This survey was approved by the CNIL [French authority of data protection (“Commission Nationale Informatique et Libertés” No. 2003X727AU)] and the CNIS [French national council for statistical information (“Conseil National de l’Information Statistique”)]. INCA2 remains the most recent version of an available French population-based survey providing dietary data. A detailed survey methodology is available elsewhere (17, 18). This study focused on the adult population (*n* = 2,624). Individuals who completed the report less than 7 days were excluded as well as under-reporters identified using Black equations (19), leading to a final sample of 1,863 individuals (1,111 women and 752 men).

Individual socioeconomic variables were collected using a self-reported questionnaire and a face-to-face questionnaire. Food intakes were collected using a 7-day record in which each individual reported all foods and beverages consumed at home or outside on seven consecutive days, during three meals and three snacking occasions. “REF_FOOD” intakes were the amount in g/day of each individual food consumed by each individual from their dietary record. The CIQUAL 2013 French food composition database (20) was used to estimate “REF_NUT” intakes.

### Development of the FFQ

The FFQ used in this study was designed to assess food and nutrient intakes of adults during the previous month. The list of items was developed by experts, according to the nutritional content of foods and the type of foods (raw or cook, liquid, etc.) using data from the national dietary survey INCA2. Each item of the FFQ is a combination of individual foods (e.g., the item “fatty fish” is the combination of “cooked salmon,” “cooked trout,” “sardine in vegetal oil,” and others) from the list of foods consumed in the INCA2 study. The quantitative French FFQ contains 94 items. Portion sizes are requested for 50 items using units (one egg, two eggs), manufacture’s containers (one can of soft drink, etc.), or household measures (one teaspoon, two teaspoons, etc.). The number of different portion sizes proposed varies across items. For the items “raw vegetables,” “cooked vegetables,” “pasta/rice/semolina,” “whole grains starches,” and “legumes,” the respondent can choose a frequency for each different portion size proposed (1/4 of plate, 1/2 plate, a whole plate) to take into account within-person variation in portion sizes if respondent consumed these foods as a side dish as well as a main dish. For breads, frequencies and portion sizes are requested by moment of consumption. Portion sizes are not requested for 44 items, for which there are no simple units or household measures, or, for which portion sizes varied slightly in the population of interest (e.g., yogurt). Frequencies and portion sizes of beer, wine, and strong alcohol are also requested. An additional question asks whether the respondent adds salt in his/her plate for each meal.

Sex-specific item-composition databases were developed as follows. The nutritional composition of each item was derived from a wider list of corresponding foods from the food composition database associated with the INCA2 survey. The list of foods used to derive the nutrient content of each item was selected, according to the number of foods related to each item, and the frequency of consumption of each food, avoiding to take into account too peculiar and rarely consumed foods. For the items which were related to more than 25 different foods, half of foods related to this item were selected, as being the most frequently consumed foods. For the items related between 8 and 25 foods, 75% of the related foods were taken into account to derive the nutrient content of the item, and 100% for the items which were related to less than 8 foods. The most frequently consumed foods were identified using the percentage of consumers among adults from the INCA2 survey. For each item and sex, the nutritional composition was calculated as a mean weighted by the intake of its related foods by adults from the INCA2 survey. Portion sizes were assigned based on the manufacturer’s weights or household measures for items for which portion sizes were requested. For the others items, a unique sex-specific portion size was assigned as the median quantity eaten daily among French adults in the INCA2 survey.

### “FFQ_FOOD” and “FFQ_NUT” Intakes

For each INCA2 individual and each item, “FFQ” frequency was simulated by calculating the number of times an item was declared in his/her INCA2 dietary record. For instance, the frequency of consumption of the item “all-season fresh fruits” for an individual who has declared to have consumed every day an apple, and two times a banana during the week of data collection, was nine times a week.

“FFQ_FOOD” intakes in g/day have been estimated by multiplying the simulated “FFQ” frequencies by portion sizes. Individual portion sizes were used for the 50 items for which portion sizes were requested. The individual portion size was chosen as the closest portion size that an individual could chose in the FFQ, based on his/her own individual median portion size. For instance, an individual for whom 50% of his/her reported intake of eggs was 125 g during the week of interview was attributed a portion size of 120 g in the “eggs” item of the FFQ (twice the weight of a standard egg at 60 g).

“FFQ_NUT” intakes were calculated by multiplying the “FFQ_FOOD” intakes by the sex-specific item-composition database.

### “ITEM_NUT” Intakes

In order to assess only the impact of the item-composition database on nutrient intakes, “ITEM_NUT” intakes were calculated for each individual by multiplying the exact amounts consumed of items, estimated from the self-reported dietary record (i.e., sum of the intake of each individual foods related to the item) by the sex-specific item-composition database.

### Statistical Analysis

Each food and item were categorized into 8 food groups and 34 food subgroups. Food categorization is presented in Table S1 in Supplementary Material.

For each food group and subgroup, “REF_FOOD” and “FFQ_FOOD” mean intakes (with the exclusion of non-consumers within food groups and subgroups) were estimated and compared, to assess the impact of using portion sizes instead of real quantities, using mixed generalized linear model with repeated measures. Measurement errors were quantified through calculating the variations in absolute values between “FFQ_FOOD” and “REF_FOOD,” expressed in percentage of “REF_FOOD” intakes by food groups and subgroups. A threshold of 5% of variation (in %) was chosen to identify consumers with an underestimation (i.e., variation below −5%) or overestimation (i.e., variation above 5%), for each food group and subgroup. Measurement errors were compared between individuals with over- and underestimation by generalized linear model by food groups and subgroups. The direction of measurement error was visualized for each food group by plotting mean food intake variations (in %) against deciles of “FFQ_FOOD” intakes (excluding non-consumers). Relative agreements between “FFQ_FOOD” and “REF_FOOD” intakes were assessed by food groups and subgroups, using cross-classification into quartiles of food intakes, weighted Kappa coefficients, and Spearman correlation.

“REF_NUT,” “ITEM_NUT,” and “FFQ_NUT” mean daily total energy and macronutrients in % energy, as well as the intakes of water, fiber, docosahexaenoic acid (DHA), eicosapentaenoic acid (EPA), α-linolenic and linoleic acids, 11 vitamins, and 10 minerals (with the exclusion of alcoholic beverages) were estimated. Pairwise comparisons were performed between “REF_NUT,” “ITEM_NUT,” and “FFQ_NUT” intakes using mixed generalized linear model with repeated measures, first to identify the impact on nutrient intakes of using the item-composition database by comparing “REF_NUT” and “ITEM_NUT” intakes and then to identify the impact of the use of average portion sizes by comparing “ITEM_NUT” with “FFQ_NUT” intakes. Measurement errors in nutrient intakes were assessed for each nutrient by calculating mean variations in absolute values between “ITEM_NUT” or “FFQ_NUT” and “REF_NUT” intakes, in percentage of “REF_NUT” intakes. For each nutrient, measurement errors were compared, using generalized linear models, between individuals with over- and underestimations identified as described earlier. Variations (in %) between “FFQ_NUT” and “REF_NUT” energy and macronutrient intakes were plotted against deciles of “FFQ_NUT” intakes. The relative agreements between “REF_NUT” and “FFQ_NUT” intakes were assessed using cross-classification and weighted Kappa coefficients. Weighted Kappa coefficients (one per nutrient) were plotted in descending order. For each nutrient, the association between “REF_NUT” intakes and the two other estimates was also tested using Spearman correlation coefficient.

All analyses were adjusted on “REF_NUT” total energy intakes, age and gender, and were performed with SAS Version 9.4. An α level of 1% was used for all statistical tests.

## Results

### Comparison between “REF_FOOD” and “FFQ_FOOD” Intakes

On average, “FFQ_FOOD” total food intake was lower than “REF_FOOD” total food intake (−198 g/day), with a measurement error of 10.7% (Table 1). “FFQ_FOOD” total food intake was considered as underestimated for 56.8% of consumers and overestimated for 12.7%, with measurement errors of 15.0% and 11.1%, respectively.

**Table 1:** “REF_FOOD” and “FFQ_FOOD” mean intakes,^a^ and measurement errors between “REF_FOOD” and “FFQ_FOOD” intakes among all consumers and among consumers with over- or underestimation.

		Food intakes, in g/j								Measurement errors^b^									
		REF_FOOD		FFQ_FOOD			Among all consumers			Individuals with underestimation^c^				Individuals with overestimation^d^					Individuals with a good estimation^e^
									
Food group	*N*^f^	Mean	SD	Mean	SD	*p* Value^g^	Mean	SD	Median	*N*, %	Mean	SD	Median	*N*, %	Mean	SD	Median	*p* Value^h^	*N*, %
Total quantity	1,863	2,550.0	751.8	2,351.1	718.0	<0.001	10.7	8.4	8.9	56.8	15.0	7.9	13.0	12.7	11.1	5.6	9.4	<0.001	30.5
Fruits and vegetables	1,850	291.7	188.8	296.0	172.3	0.018	21.3	38.5	14.4	32.2	17.3	10.4	14.5	48.9	31.3	52.1	21.1	<0.001	19.0
Vegetables	1,839	130.3	80.3	147.0	85.2	<0.001	24.9	47.2	13.8	16.2	12.6	6.3	10.6	62.0	36.1	56.9	22.6	<0.001	21.8
Fresh and processed fruits	1,677	176.7	142.6	163.6	118.1	<0.001	33.7	134.2	21.2	48.0	24.9	14.0	22.3	39.3	54.5	211.7	29.7	<0.001	12.7
Nuts and oilseeds	502	7.5	9.2	5.7	6.5	<0.001	59.7	111.0	41.0	50.8	43.0	20.5	40.0	37.5	100.9	171.1	60.0	<0.001	11.8
Starches	1,862	242.5	123.6	235.7	107.7	<0.001	9.6	9.0	7.4	34.4	12.9	8.2	10.5	30.9	14.1	9.5	11.3	0.006	34.7
Breads	1,829	113.5	83.1	106.9	64.6	<0.001	10.3	15.1	6.1	28.6	16.8	12.0	13.0	27.6	16.8	22.0	11.3	0.931	43.8
Starches and legumes	1,746	74.1	57.2	71.0	50.9	<0.001	16.6	25.2	11.1	37.7	18.6	8.7	16.7	24.3	39.0	39.8	25.0	<0.001	38.0
Potatoes	1,681	62.9	46.6	66.5	52.0	<0.001	15.8	18.7	11.8	22.8	17.7	9.2	16.7	43.1	27.1	21.1	25.0	<0.001	34.1
Cereals for breakfast	318	27.8	27.5	23.7	17.7	<0.001	44.3	52.4	33.9	47.5	34.0	17.6	32.5	37.7	74.6	71.3	50.0	<0.001	14.8
Meat/fish/eggs and substitutes	1,861	158.2	69.7	141.3	54.2	<0.001	19.6	16.6	16.6	55.7	22.2	11.9	20.3	29.1	23.5	22.0	18.0	0.078	15.2
Eggs	1,172	23.2	16.3	23.7	15.6	<0.001	27.5	127.4	14.9	23.0	18.4	10.1	15.9	53.5	43.1	172.5	14.9	0.015	23.5
Fish	1,482	34.3	25.0	26.9	18.4	<0.001	49.1	68.1	37.0	60.8	37.9	18.0	37.5	33.5	77.5	108.9	46.1	<0.001	5.7
Meats	1,819	80.6	49.7	73.9	37.2	<0.001	25.2	28.3	19.4	46.2	24.1	13.7	21.6	38.8	35.3	39.3	25.0	<0.001	15.0
Deli meats	1,689	35.6	27.2	30.6	20.9	<0.001	36.6	67.7	24.7	55.8	29.4	17.5	27.5	34.5	58.0	109.2	30.8	<0.001	9.7
Offals	422	21.7	13.0	20.6	10.2	0.005	49.3	82.0	25.0	48.3	28.6	14.9	23.3	41.2	85.6	117.1	35.3	<0.001	10.4
Protein substitutes	17	28.8	22.1	26.1	15.3	0.163	22.5	24.6	25.0	41.2	26.1	6.2	25.0	23.5	50.0	33.3	33.3	0.095	35.3
Mixed dishes and sandwiches	1,844	206.7	138.4	183.4	113.0	<0.001	21.8	23.3	17.4	56.2	22.6	12.6	21.1	27.9	31.4	36.1	20.8	<0.001	15.8
Soups	1,003	160.5	126.6	139.6	101.8	<0.001	21.1	28.6	20.0	51.0	25.9	13.6	25.0	23.0	33.7	49.4	20.0	0.001	25.9
Mixed dishes	1,563	80.2	64.4	72.4	51.7	<0.001	40.0	56.8	28.8	51.8	29.7	15.3	28.9	36.9	66.6	84.3	40.6	<0.001	11.4
Sandwiches, snacks, and salt pastries	1,568	60.4	55.1	54.3	45.2	<0.001	27.5	61.3	15.5	49.5	22.4	15.4	17.7	33.5	48.0	100.3	22.2	<0.001	17.0
Dairy products and substitutes	1,842	203.7	161.1	194.6	144.2	<0.001	15.1	20.2	9.7	43.8	17.6	11.8	13.5	26.0	26.4	31.7	16.7	<0.001	30.3
Milk	916	168.7	152.2	167.8	128.8	0.650	59.9	163.5	12.9	46.0	14.6	8.4	10.5	39.0	136.1	243.0	32.2	<0.001	15.1
Yogurt	1,462	101.9	77.4	97.3	65.9	<0.001	13.6	48.3	3.3	21.8	24.6	15.3	20.0	24.2	33.0	92.7	16.7	0.037	54.0
Cheese	1,728	36.1	27.7	32.2	27.4	<0.001	20.9	17.1	19.4	57.9	25.6	13.2	24.5	25.9	22.1	21.3	18.4	<0.001	16.1
Dairy substitutes	100	91.9	92.9	67.5	53.6	<0.001	37.4	69.8	15.0	40.0	40.6	21.1	38.7	19.0	110.8	130.3	30.7	0.005	41.0
Sweet products	1,825	120.0	77.3	104.5	66.1	<0.001	22.3	20.2	18.1	58.0	25.5	15.1	22.3	27.5	26.5	27.4	19.1	0.547	14.6
Ice creams and dairy desserts	1,074	41.8	40.1	39.6	32.0	<0.001	44.4	354.8	18.0	43.2	22.6	14.1	20.0	42.4	81.2	543.1	27.8	0.021	14.4
Cakes, tarts, and pastries	1,683	73.5	53.1	64.2	45.0	<0.001	34.7	50.0	25.0	53.4	30.7	17.0	28.0	34.3	52.7	78.2	30.4	<0.001	12.4
Biscuits and sweets	1,684	30.0	26.3	23.8	19.5	<0.001	24.7	24.3	18.8	57.7	31.3	20.0	27.1	23.1	27.6	31.7	21.3	0.123	19.2
Water and other beverages	1,860	1,433.8	650.6	1,163.9	611.5	<0.001	22.3	16.9	18.7	71.9	27.8	16.2	24.8	13.3	15.0	8.4	13.1	<0.001	14.8
Water	1,791	800.3	553.2	789.5	568.3	0.011	25.7	351.9	14.1	40.7	20.3	11.7	17.9	39.2	43.2	561.8	18.6	0.200	20.1
Hot drinks	1,699	439.6	331.8	313.9	247.4	<0.001	28.2	33.9	26.2	76.7	33.5	17.1	32.4	11.1	20.2	85.8	11.5	<0.001	12.2
Light drink	218	122.8	149.2	119.9	152.9	0.101	26.4	132.6	17.5	40.4	22.2	10.6	17.5	36.2	47.8	218.8	24.5	0.315	23.4
Sweet drinks	640	152.2	232.9	147.3	214.8	0.030	60.3	264.3	17.5	36.4	23.5	12.5	17.5	43.0	120.0	395.4	32.0	0.001	20.6
Fruit juices	905	113.1	96.1	107.2	102.5	<0.001	32.3	154.3	20.0	55.4	25.4	10.4	26.7	36.8	49.2	253.2	18.8	0.032	7.8
Fats and condiments	1,858	43.5	23.0	42.5	22.1	<0.001	14.0	12.6	10.6	38.5	18.4	12.1	15.5	35.6	17.5	12.6	13.9	0.599	25.9
Animal fats	1,627	15.5	13.2	14.9	12.8	<0.001	14.1	13.7	11.1	39.8	20.2	11.7	17.6	29.3	19.5	14.2	16.0	<0.001	30.9
Vegetal fats	1,703	16.7	12.8	17.4	13.6	<0.001	19.1	18.3	16.8	34.1	19.3	9.9	19.4	46.7	26.1	21.8	25.0	<0.001	19.2
Hot sauces	1,106	11.3	10.7	9.4	6.5	<0.001	54.9	83.1	33.3	44.1	40.8	19.2	38.5	36.8	100.2	120.3	73.1	<0.001	19.1
Cold sauces	1,327	11.1	9.4	11.0	9.1	0.176	14.2	13.7	11.1	35.3	16.0	10.4	15.0	36.3	23.0	13.9	25.0	<0.001	28.4
Salt	438	1.0	1.3	0.8	0.8	<0.001	14.8	25.7	0.0	24.2	42.6	19.1	45.0	7.5	58.7	35.0	54.3	0.001	68.3

*^a^“REF_FOOD” and “FFQ_FOOD” mean food intakes were calculated among consumers by food group and subgroups*.

*^b^Measurement error was assessed by food groups and subgroups through the variation in absolute values between “FFQ_FOOD” and “REF_FOOD” intakes, expressed in percentage of “REF_FOOD” intake*.

*^c^Individuals for which the variation in percentage was below or equal to −5%*.

*^d^Individuals for which the variation in percentage was above or equal to 5%*.

*^e^Individuals for which the variation in percentage was between −4.99 and 4.99% (measurement error below 5%)*.

*^f^Number of consumers*.

*^g^p Value of the mixed generalized linear model with repeated measures, testing differences between “FFQ_FOOD” and “REF_FOOD” intakes, adjusted on sex, age, and “REF_NUT” total energy*.

*^h^p Value of the generalized linear model, testing differences in the relative variation between individual with over- or underestimation of food intakes, adjusted on sex, age, and “REF_NUT” total energy intake*.

**Figure 1:**
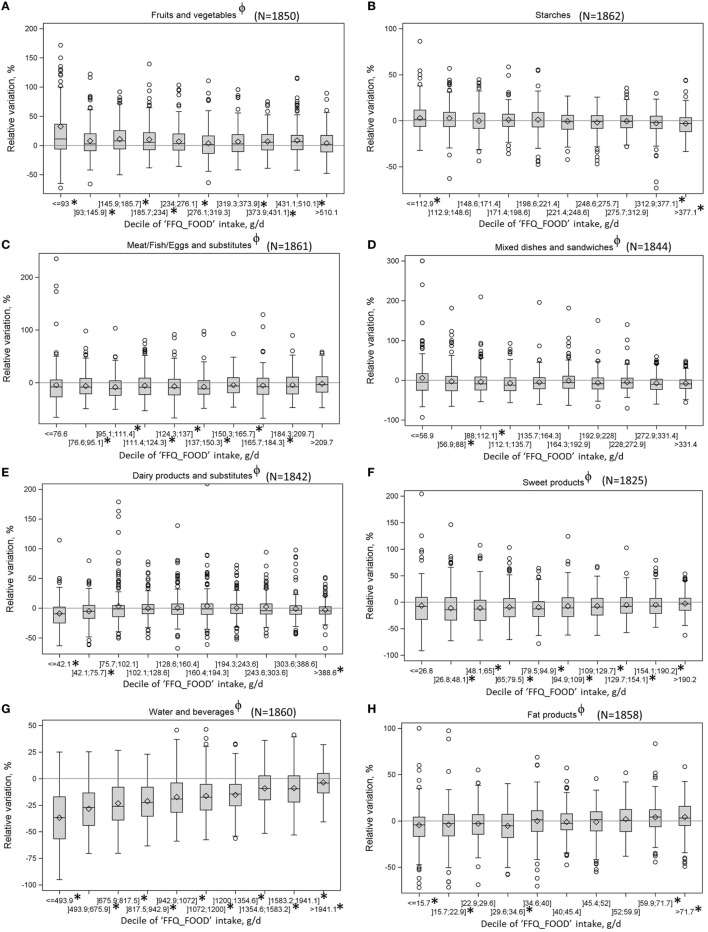
Variations between “FFQ_FOOD” and “REF_FOOD” intakes (in %) by deciles of “FFQ_FOOD” intakes^a^, among consumers of each food group^b^ **(A–H)**. ^a^A negative variation indicates an underestimation, the symbol ϕ means a significant *p*. for linear trend and the symbol * means a variation significantly different from 0. ^b^The maximum y-axis has been set to 200% for fruits and vegetables, dairy products, and sweet products because of extreme values.

On average, “FFQ_FOOD” mean intakes of all food groups were significantly different from “REF_FOOD” mean intakes, except for fruits and vegetables. Measurement error ranged from 9.6% for starches to 22.3% for sweet products and water and other beverages food groups (Table 1). “FFQ_FOOD” tended to overestimate fruits and vegetables intakes (48.9% of individuals with an overestimation and 32.2% with an underestimation) and underestimate the other food group intakes, except starches for which the percentage of individuals with an error measurement lower than 5% was the highest (34.7%) and the variation (in %) was close to 0 for almost all deciles of “FFQ_FOOD” intakes (*p* for trend not significant) (Figure 1). Water and other beverages food groups had the highest percentage of individuals with underestimation (71.9%), with a mean measurement error of 27.8%.

At food subgroup level, no significant differences were found between “FFQ_FOOD” and “REF_FOOD” intakes for protein substitutes, milk, water, light drinks, sweet drinks, and cold sauces subgroups (Table 1). Measurement errors between “FFQ_FOOD” and “REF_FOOD” intakes were above 40% for nuts and oilseeds, cereals for breakfast, fish, offal, mixed dishes, milk, ice cream and dairy desserts, sweet drinks, and hot sauces subgroups. However, SD values were high and medians were much lower than the means, indicating that the high measurement errors were steered by the values reached by specific consumers. For salt, yogurt, breads, dairy substitutes, and starches and legumes, measurement error was below 5% for more than 30% of consumers (68.3, 54, 43.8, 41, 38, and 34.7% of consumers, respectively). Vegetables, eggs, vegetal fats, potatoes, sweet drinks, and cold sauces subgroups were mainly overestimated (percentage of consumers with overestimation greater than percentage of consumers with underestimation or with a low measurement error), whereas the remaining food subgroups were mainly underestimated. The highest mean measurement errors among food groups with a high proportion of overestimation were 120% for sweet drinks, followed by eggs (43.1%) and vegetables (36.1%). Among food subgroups with high proportion of underestimation, the highest mean measurement errors were for nuts and oilseeds (43%), followed by hot sauces (40.8%) and fish (37.9%). Average measurement errors were not different between consumers with an overestimation and those with an underestimation for bread, eggs, protein substitutes, yogurt, ice cream and dairy desserts, biscuit and sweets, water, light drink, and fruit juices subgroups.

Spearman correlation coefficients and cross-classification into quartiles between “REF_FOOD” and “FFQ_FOOD” food group and subgroup intakes are presented in Table S2 in Supplementary Material. The lowest Spearman correlation coefficient was for meat/fish/eggs and substitutes and water and other beverages (0.82). The percentage of individuals with an “exact agreement” was above 60% for 28 food subgroups (out of 34) and for most food groups (dairy products, fats and condiments, fruits and vegetables, mixed dishes and sandwiches, and sweet products). The percentage of individuals with “extreme disagreement” was low for all food groups and subgroups with the highest values for fats and condiment food group (4.3%) and hot sauces subgroup (1.4%).

### Comparison “REF_NUT,” “ITEM_NUT,” and “FFQ_NUT” Intakes

#### Impact on Nutrient Intakes Estimates of Using the Item-Composition Database

For all nutrients except free sugars (% energy), “ITEM_NUT” intakes were not significantly different from “REF_NUT” intakes (Table 2). Variations in absolute values between “ITEM_NUT” and “REF_NUT” intakes (in %) ranged from 1.2% for water to 42.5% for EPA. The highest measurement error (above 15%) were found for EPA (42.4%), DHA (39.7%), vitamin A (36.4%), free sugars (in % energy) (33%), vitamin B-12 (27.8%), vitamin D (23.1%), α-linolenic acids (20.3%), vitamin C (18.9%), copper (18.3%), and iodine (16.8%).

**Table 2:** “REF_NUT,” “ITEM_NUT,” and “FFQ_NUT” mean daily total energy and nutrient intakes and measurement errors^a^ between “ITEM_NUT” or “FFQ_NUT” with “REF_NUT” nutrient intakes.

	Nutrient intakes									Measurement errors					
	REF_NUT		ITEM_NUT		FFQ_NUT					Between ITEM_NUT and REF_NUT			Between FFQ_NUT and REF_NUT		
								
Nutrient	Mean	SD	Mean	SD	Mean	SD	*p* Value^b^	*p* Value^c^	*p* Value^d^	Mean	SD	Median	Mean	SD	Median
Energy (kcal/day)	2,075.8	580.4	2,075.9	579.6	1,917.0	500.5	0.960	<0.001	<0.001	3.0	2.5	2.5	10.6	8.1	9.0
Energy (kJ/day)	8,699.4	2,433.7	8,699.8	2,431.4	8,033.4	2,097.2	0.960	<0.001	<0.001	3.0	2.5	2.5	10.6	8.1	9.0
Proteins (% AET)	16.5	2.9	16.5	2.8	16.3	2.5	0.474	<0.001	<0.001	3.9	3.2	3.0	7.3	5.9	6.0
Carbohydrates (% AET)	42.7	6.2	42.6	6.1	42.8	6.0	0.241	0.003	0.071	3.0	2.5	2.5	5.4	4.7	4.3
Total sugars (% AET)	16.8	5.4	16.8	5.3	16.4	5.1	0.980	<0.001	<0.001	6.2	5.8	4.7	10.5	8.8	8.5
Free sugars (% AET)	8.2	4.8	9.4	5.0	9.0	4.8	<0.001	<0.001	<0.001	33.0	92.0	15.6	35.7	109.8	17.5
Total fat (% AET)	38.5	5.8	38.6	5.4	38.5	5.5	0.408	0.242	0.749	4.0	3.5	3.1	6.1	5.6	4.7
Saturated fat (% energy)	14.8	3.1	14.8	2.8	14.7	2.9	0.067	<0.001	<0.001	6.3	5.8	4.9	8.4	7.4	6.6
Monounsaturated fatty acids (% AET)	13.6	3.0	13.7	2.8	13.7	3.0	0.322	0.186	0.106	6.1	5.2	4.9	8.8	7.6	7.1
Poly-unsaturated fatty acids (% AET)	5.7	2.0	5.7	1.9	5.8	2.0	0.432	<0.001	<0.001	10.1	11.2	7.3	12.2	11.7	9.2
Fiber (g/day)	18.1	6.4	18.1	6.2	17.3	5.5	0.699	<0.001	<0.001	7.3	6.6	5.5	13.0	10.2	10.7
Water (g/day)	2,115.8	700.3	2,115.8	699.7	1,951.4	677.0	0.930	<0.001	<0.001	0.5	0.5	0.4	11.4	9.1	9.4
Eicosapentaenoic acid (mg/day)	106.0	99.3	106.8	94.1	84.3	62.0	0.542	<0.001	<0.001	42.4	48.9	29.0	41.7	44.2	33.6
Docosahexaenoic acid (mg/day)	143.9	131.8	144.5	124.4	114.9	83.4	0.703	<0.001	<0.001	39.7	69.1	23.2	47.3	63.5	34.7
α-Linolenic acid (g/day)	1.1	0.6	1.1	0.5	1.0	0.5	0.669	<0.001	<0.001	20.3	19.6	15.7	20.8	17.4	16.5
Linoleic acid (g/day)	11.6	5.2	11.6	4.9	11.0	5.1	0.705	<0.001	<0.001	11.0	12.4	7.8	15.6	13.1	12.8
Sodium (g/day)	3,039.3	1,056.4	3,037.1	1,017.4	2,768.2	813.9	0.741	<0.001	<0.001	6.8	5.6	5.3	12.7	10.0	10.4
Potassium (mg/day)	2,865.2	799.6	2,866.2	789.4	2,590.2	656.3	0.777	<0.001	<0.001	4.4	3.9	3.6	12.6	9.6	10.6
Magnesium (mg/day)	306.9	97.4	306.9	86.9	273.2	68.8	0.985	<0.001	<0.001	8.9	7.6	7.2	13.8	10.8	11.4
Calcium (mg/day)	886.9	311.6	888.8	286.7	822.5	268.6	0.530	<0.001	<0.001	10.6	9.5	7.9	14.1	11.2	11.5
Copper (mg/day)	1.5	0.8	1.5	0.5	1.4	0.4	0.816	<0.001	<0.001	18.3	21.3	11.2	19.6	18.9	14.0
Iron (mg/day)	11.3	4.1	11.2	3.7	10.3	3.0	0.264	<0.001	<0.001	11.4	9.6	9.4	15.5	12.3	12.7
Phosphorus (mg/day)	1,221.2	340.9	1,222.8	333.6	1,116.8	286.4	0.477	<0.001	<0.001	5.8	4.9	4.6	12.3	9.1	10.6
Iodine (g/day)	129.8	51.8	129.0	40.3	118.1	36.6	0.307	<0.001	<0.001	16.8	16.4	12.9	17.7	16.2	14.1
Zinc (g/day)	10.2	3.4	10.2	3.1	9.4	2.5	0.813	<0.001	<0.001	9.1	7.8	6.8	14.4	10.9	12.2
Selenium (g/day)	81.7	32.9	81.9	23.9	70.6	18.5	0.636	<0.001	<0.001	13.6	14.2	9.3	17.9	14.4	14.6
Vitamin A (ER/day)	1,127.8	793.0	1,126.5	525.6	1,074.9	481.5	0.926	<0.001	<0.001	36.4	43.8	24.4	35.0	37.3	25.2
Vitamin C (mg/day)	92.6	53.7	92.7	48.9	86.6	44.7	0.886	<0.001	<0.001	18.9	26.8	12.3	23.2	23.1	17.6
Vitamin D (μg/day)	2.6	1.4	2.6	1.2	2.3	0.9	0.351	<0.001	<0.001	23.1	24.8	16.5	26.6	23.1	22.0
Vitamin E (mg/day)	12.3	5.9	12.3	5.6	11.9	6.1	0.479	<0.001	<0.001	11.5	11.4	8.5	15.8	12.9	13.1
Thiamin (mg/day)	1.2	0.4	1.2	0.4	1.1	0.3	0.513	<0.001	<0.001	10.3	9.2	8.1	14.2	11.5	11.8
Riboflavin (mg/day)	1.7	0.6	1.6	0.5	1.5	0.5	0.716	<0.001	<0.001	10.1	9.3	7.4	14.4	11.3	12.0
Niacin (mg/day)	18.1	6.5	18.2	6.0	16.2	4.5	0.382	<0.001	<0.001	11.0	9.5	8.4	17.1	13.2	14.7
Panthotenic acid (mg/day)	5.0	1.6	5.0	1.5	4.6	1.2	0.966	<0.001	<0.001	9.4	8.7	7.2	13.6	10.9	11.0
Vitamin B-6 (mg/day)	1.7	0.6	1.7	0.5	1.5	0.4	0.569	<0.001	<0.001	9.4	8.1	7.5	14.4	11.3	12.1
Folate (μg/day)	280.1	97.4	279.8	87.4	267.5	80.1	0.719	<0.001	<0.001	11.6	10.1	9.1	14.8	11.6	12.4
Vitamin B-12 (μg/day)	5.7	3.8	5.7	2.8	5.1	2.4	0.604	<0.001	<0.001	27.8	36.6	18.8	28.3	31.5	20.4

*^a^Measurement error was assessed by nutrient through the variation in absolute values between “ITEM_NUT” or “FFQ_NUT” and “REF_NUT” intake, expressed in percentage of “REF_NUT” intake*.

*^b^*p* Value of the mixed generalized linear model with repeated measures, testing differences between “ITEM_NUT” and “REF_NUT” intakes, adjusted for sex, age, and “REF_NUT” total energy (except for energy variable)*.

*^c^*p* Value of the mixed generalized linear model with repeated measures, testing differences between “FFQ_NUT” and “ITEM_NUT” intakes, adjusted for sex, age, and “REF_NUT” total energy (except for energy variable)*.

*^d^*p* Value of the mixed generalized linear model testing differences between “FFQ_NUT” and “REF_NUT” intakes, adjusted for sex, age, and “REF_NUT” total energy (except for energy variable)*.

#### Impact on Nutrient Intakes Estimates of Using Portion Sizes

For total fat (in % energy) and monounsaturated fatty acids (in % energy), “ITEM_NUT” intakes were not significantly different from “FFQ_NUT” intakes (Table 2). For remaining nutrients, “FFQ_NUT” mean intake was always lower than “ITEM_NUT” mean intake, except for carbohydrates (in % energy) and poly-unsaturated fatty acids (in % energy).

#### Overall Impact on Nutrient Intakes Estimates of the Inherent Structure of the Questionnaire

**Figure 2:**
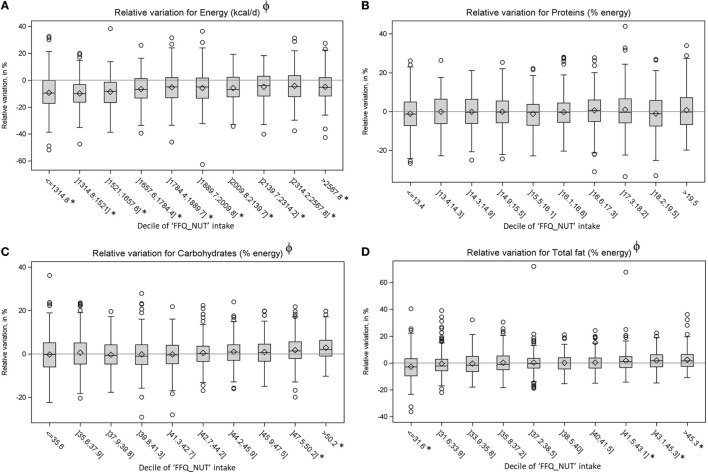
Variations between “FFQ_NUT” and “REF_NUT” total energy intake **(A)** and macronutrients (in % energy) **(B–D)** by decile of “FFQ_NUT” intakes^a^. ^a^A negative variation indicates an underestimation, the symbol ϕ means a significant *p*. for linear trend and the symbol * means a variation significantly different from 0.

Mean energy intake was 8699 and 8033 kJ/d (2,075 and 1,917 kcal/day) for “REF_NUT” and “FFQ_NUT,” respectively, leading to an underestimation of 666 kJ/d (158 kcal) (Table 2). “FFQ_NUT” energy intake was underestimated for 55% individuals, with a mean measurement error of 14.7% (Table 3). “FFQ_NUT” energy intake was underestimated whatever the decile of “FFQ_NUT” energy intake, with a negative variation which came closer to 0 with increasing “FFQ_NUT” intakes (*p* for trend < 0.01) (Figure 2).

**Table 3:** Measurement errors^a^ between “FFQ_NUT” and “REF_NUT” total energy and nutrient intakes among individuals with over- and underestimation (*N* total = 1,863).

	Individuals with underestimation^b^				Individuals with overestimation^c^					Individuals with a good estimation^d^
				
Nutrient	*N*, %	Mean	SD	Median	*N*, %	Mean	SD	Median	*p* Value^e^	*N*, %
Energy (kcal/day)	55.3	14.7	7.5	13.1	14.9	11.3	5.8	9.7	<0.001	29.8
Energy (kJ/day)	55.6	14.7	7.5	13.1	14.9	11.3	5.8	9.7	<0.001	29.5
Proteins (% AET)	29.8	10.7	4.8	9.6	26.8	11.4	5.7	9.6	0.050	43.4
Carbohydrates (% AET)	19.2	8.9	3.8	7.8	24.4	9.8	4.6	8.5	0.002	56.4
Total sugars (% AET)	38.4	13.7	7.3	11.9	30.2	14.6	9.4	12.0	0.137	31.3
Free sugars (% AET)	26.4	18.9	12.9	14.6	57.8	52.4	141.7	27.1	<0.001	15.8
Total fat (% AET)	23.7	9.2	3.9	8.1	23.8	11.0	6.9	9.1	<0.001	52.5
Saturated fat (% energy)	32.5	11.4	5.6	9.8	29.0	13.1	8.5	10.4	<0.001	38.4
Monounsaturated fatty acids (% AET)	29.6	11.5	5.3	10.1	32.6	13.6	8.5	11.4	<0.001	37.8
Poly-unsaturated fatty acids (% AET)	31.1	13.7	7.0	11.9	39.7	18.3	14.1	14.2	<0.001	29.3
Fiber (g/day)	45.1	16.1	8.7	14.3	31.0	16.6	10.6	13.7	0.191	23.9
Water (g/day)	56.0	15.9	8.7	13.7	15.3	11.7	6.3	9.9	<0.001	28.7
Eicosapentaenoic acid (mg/day)	54.5	16.9	9.6	14.7	19.9	14.0	7.8	12.1	<0.001	25.6
Docosahexaenoic acid (mg/day)	55.1	34.4	17.1	33.8	37.9	59.5	63.7	41.6	<0.001	7.0
α-Linolenic acid (g/day)	55.2	35.6	18.0	34.8	36.8	74.6	95.5	46.1	0.003	8.1
Linoleic acid (g/day)	45.0	23.7	14.4	20.4	39.5	24.7	19.2	19.6	0.260	15.5
Sodium (g/day)	50.3	18.1	9.6	16.4	29.3	20.2	16.7	15.2	0.003	20.4
Potassium (mg/day)	60.3	16.5	8.8	14.8	14.7	13.6	8.0	11.4	<0.001	25.0
Magnesium (mg/day)	58.8	18.2	10.3	16.2	17.8	13.9	8.5	11.3	<0.001	23.4
Calcium (mg/day)	51.0	18.1	10.2	15.8	24.8	17.0	11.0	14.2	0.065	24.2
Copper (mg/day)	46.6	21.2	15.1	16.7	35.5	26.2	22.6	18.7	<0.001	17.9
Iron (mg/day)	50.9	19.3	11.0	17.1	27.9	18.4	12.7	14.8	0.293	21.3
Phosphorus (mg/day)	57.3	16.2	8.5	14.8	17.8	13.3	6.8	11.4	<0.001	25.0
Iodine (g/day)	49.6	21.4	12.7	19.0	30.3	21.7	20.1	16.8	0.585	20.1
Zinc (g/day)	50.8	18.3	10.1	16.1	27.5	16.7	10.1	13.9	0.035	21.7
Selenium (g/day)	57.5	22.2	13.5	19.3	23.6	19.6	14.2	15.6	0.001	18.8
Vitamin A (ER/day)	39.9	28.4	16.2	25.7	49.3	47.5	46.9	33.6	<0.001	10.8
Vitamin C (mg/day)	46.8	22.8	13.0	19.8	38.5	31.7	30.7	22.5	<0.001	14.7
Vitamin D (μg/day)	52.1	27.4	14.5	25.4	35.3	34.0	30.7	24.5	<0.001	12.6
Vitamin E (mg/day)	44.8	18.9	10.9	16.4	34.1	19.7	14.0	15.6	0.342	21.0
Thiamin (mg/day)	50.2	18.4	10.8	16.1	26.8	16.6	10.7	13.8	0.002	23.0
Riboflavin (mg/day)	56.0	18.7	10.6	16.7	22.0	15.5	10.1	12.6	<0.001	22.0
Niacin (mg/day)	55.4	20.6	11.5	18.3	26.4	19.9	13.9	16.0	0.429	18.3
Panthotenic acid (mg/day)	52.5	17.9	10.0	15.7	23.0	15.7	10.2	12.2	0.001	24.4
Vitamin B-6 (mg/day)	51.1	17.9	10.0	15.8	26.6	17.6	11.7	14.2	0.707	22.3
Folate (μg/day)	45.4	17.4	9.9	15.2	34.6	18.5	12.2	15.3	0.092	20.0
Vitamin B-12 (μg/day)	48.3	26.6	15.5	23.2	39.2	38.6	43.7	25.6	<0.001	12.6

*^a^Measurement error was assessed by nutrient through the variation in absolute values between “FFQ_NUT” and “REF_NUT” intake, expressed in percentage of “REF_NUT” intake*.

*^b^Individuals for which the variation in percentage was below or equal to −5%*.

*^c^Individuals for which the variation in percentage was above or equal to 5%*.

*^d^Individuals for which the variation in percentage was between −4.99 and 4.99%*.

*^e^p Value of the generalized linear model, testing differences in the relative variation between individual with over- or underestimation of food intakes, adjusted on sex, age, and “REF_NUT” total energy intake*.

For the other nutrients, no significant differences were found between “FFQ_NUT” and “REF_NUT” intakes for carbohydrates (in % energy), total fat (in % energy), and monounsaturated fatty acids (in % of energy). Measurement errors ranged from 5.4% for carbohydrate (in % energy) to 47.3% for DHA, with 15 nutrients with a measurement error above 15%. For carbohydrates (in % energy), total fat (in % energy), saturated fat (in % energy), proteins (in % energy), and monounsaturated fatty acids (in % energy), proportion of individuals with a low measurement error (i.e., below 5%) is lower than percentage of individuals with a higher one (56.4, 52.5, 43.4, 38.4, and 37.8% of consumers, respectively) (Table 3). Figure 2 shows that for carbohydrates and total fats, mean variation (in %) was significantly different from 0 only for higher deciles (and the first decile for total fat), and no difference was noticed for proteins. More individuals were considered to have an overestimation for free sugars (in % energy), poly-unsaturated fatty acids (in % energy), and vitamin A rather an underestimation or low measurement error, whereas a higher percentage of individuals were identified with an underestimation for the other nutrients. The highest measurement error among nutrients which were mostly overestimated was for free sugar (52.4%). Among nutrients with underestimation, the highest measurement errors were for α-linolenic acid (35.6%), DHA (34.4%), and vitamin D (27.4%). Average measurement errors were not different between individuals with an overestimation and those with an underestimation, for total sugars (in % energy), fiber, linoleic acids, iodine, calcium, zinc, iron, vitamins E, niacin, vitamin B-6, and folates. The variations by deciles of “FFQ_NUT” intakes for vitamins and minerals are presented in Figures S1 and S2 in Supplementary Material: they show an overall trend to underestimate micronutrient nutrient intakes, except for vitamin A, vitamin C, and vitamin B-12.

**Figure 3:**
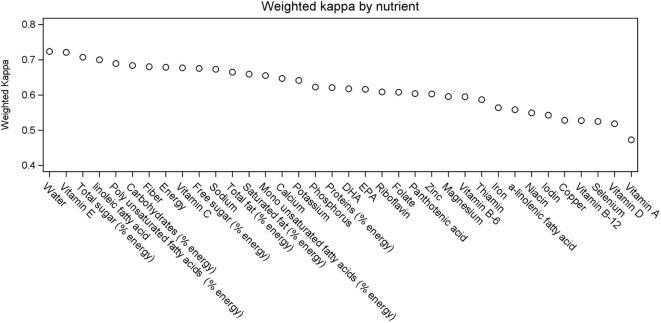
Weighted Kappa coefficients between quartiles of REF_NUT and FFQ_NUT intakes by nutrient.

Spearman correlation coefficients between “REF_NUT” and “FFQ_NUT” ranged from 0.66 to 0.90 for vitamin A intake and free sugars (in % energy), respectively (Table S3 in Supplementary Material). The percentage of individuals with an “exact agreement” was above 60% for 18 nutrients, with a minimum at 49.2% for vitamin A. The percentage of individuals with “extreme disagreement” was not above 2.1% (for vitamin A). The highest weighted Kappa coefficients between quartiles of “FFQ_NUT” and “REF_NUT” were observed for vitamin E (0.72), water (0.71), and total sugars (in % energy) and lowest for copper and vitamin B-12 (0.53), selenium and vitamin D (0.52), and vitamin A (0.47) (Figure 3). Twenty-three nutrients had a coefficient considered as “substantial agreement” (between 0.61 and 0.80) and the 13 others had a coefficient considered as “moderate agreement” (between 0.41 and 0.60).

## Discussion

This paper describes a new method for validating a FFQ, independently to the bias induced by respondent’s answers. This method was named “structural validation,” because it aims to assess the impact—on food and nutrient intakes estimates—of the inherent structure of a FFQ, especially the impact of using an aggregated food database and of using average and/or standard portion sizes. In this paper, the method was applied to a French medium-length quantitative FFQ. Results indicated an overall good structural validity, although an overall tendency to underestimate most of food groups, subgroups, and nutrient intakes was noticed. Overestimation was observed for certain food groups such as vegetables and sweet drinks, as well as certain nutrients such as free sugars. However, it was noticeable that, for some food groups, intakes were correctly estimated, notably for starches.

Measurement errors can be due to the estimation of food quantities, based on the use of portion sizes associated with each item instead of real and precise amounts. The use of portion sizes was shown to induce an overall underestimation of food intakes compared to “REF_FOOD” intakes, but on average, the magnitude of the underestimation was acceptable. The highest positive variation between “FFQ_FOOD” and “REF_FOOD” subgroups intakes was observed for the vegetables subgroup, with an average variation of +16.7 g/day, and the highest negative variation was for the hot drinks (−125.7 g/day) subgroup. The variation was above ±10 g for only six subgroups. The overall underestimation of total quantity was led by an underestimation of beverages (especially hot drinks), which are known to be difficult to assess, even with an open-ended instrument (21). To improve the accuracy of food and nutrient estimation, individual portion sizes were requested for 50 items in the present FFQ. The choice of using of a quantitative questionnaire or a qualitative questionnaire is a subject of long controversy. Some authors think that asking the respondent to report their own portion sizes does not improve significantly the validity of the questionnaire (22–25), whereas others argue that the individual portion sizes can take into account the inter-individual variability of portion sizes, which could highly differ according to gender and age (22). Nonetheless, taking into account individual portion sizes for certain items seemed to improve the estimation. In our data, the average variation in absolute values between “FFQ_FOOD” and “REF_FOOD” intakes (in %) was 25% among items for which an individual portion size was taken into account, instead of 46% among the others (data not shown).

The use of an aggregated food database can lead to measurement errors due to the dilution of the nutritional information of specific foods explaining between-person variance in nutrient intakes. But, less the aggregation is, longer the questionnaire will be. In a review, Cade et al. found that the number of food items in existing FFQs ranged from 5 to 350. There is currently no consensus about the optimal length of a questionnaire (2). Whereas the accuracy was greater using less aggregation of foods (22, 26), a food list of more than 100 items induced overestimation (6, 27, 28). In this study, the length was closed to the median identified by Cade (median length at 79 items). Without taking into account the respondent’s perception, this study showed that the use of aggregated food items did not impact the estimation of most of the nutrients except free sugars.

The whole impact of the inherent structure of the questionnaire seemed to be acceptable given the validation measurements (mean differences, cross-classification, and correlation coefficients). Yet, the FFQ showed an overall tendency to underestimate food intakes compared to REF_FOOD intakes. Positive and high measurement errors between “FFQ_FOOD” and “REF_FOOD” intakes (i.e., measured by the variation in absolute values, expressed in percentage of “REF_FOOD” intakes) were observed for specific food groups, such as vegetables, nuts and oilseed, milk, sweet drinks, or fishes. After investigation, these results were steered by some individuals who actually declared a very small intake, compared to the average portion size assigned to each item after simulation of FFQ answers. For instance, the individual food “concentrated fruit syrup” from the INCA2 dietary survey was related to the item “sweet beverages.” An overestimation of “sweet beverages” was observed for all individuals who declared “concentrated fruit syrup” in a very small amount, because of assigning a too large portion size. Similarly, some individuals declared a low intake of milk, which was found to be milk added in hot drinks (in a small portion), difficult to take into account into the simulation. This fact will be taken into account in the FFQ by using two independent questions about milk, one about milk as a drink and the other about milk added into the coffee with specific portion sizes. However, validation measures for food intakes (Table S3 in Supplementary Material) were high (high correlation coefficient and high percentage of individuals classified in the same quartile) compared to values found in the literature for French FFQs (6, 29). Regarding nutrient intakes, results indicated also an overall trend to underestimate nutrient intakes, except for some macronutrients expressed in % of energy for which “FFQ_NUT” intakes were higher than “REF_NUT” intake. Even if significant differences were observed for almost all nutrients in pairwise comparisons between nutrient intake estimates and “REF_NUT” intakes, differences between the two estimates were small for most of the nutrients, with respect to “REF_NUT” intakes. Indeed, the large sample size could partly explained a higher sensitivity for the statistical tests. Nevertheless, the numerous measures of validity for nutrient intakes (weighted Kappa values, Spearman correlation coefficient, and cross-classification into quartiles of nutrient intakes presented in Table S3 in Supplementary Material) showed a good performance of ranking individual based on their nutritional intakes, with good correlation coefficients (ranged from 0.67 to 1) compared with the range 0.5–0.8 proposed by Willett et al. (30). Finally, the comparison of results obtained in this paper with other validation studies is difficult because of different statistical methods and because we did not consider respondents’ bias. Nevertheless, this study pointed out that the inherent structure of the questionnaire (use of average portion sizes and of an aggregated food database) induced on average an underestimation of nutritional and food intakes.

This study presents limitations. First, methods based on self-reporting of food consumption, as the self-reported weekly dietary record used in this study, are prone to multiple bias, but, they are still widely used in epidemiological research. The design of a FFQ must be chosen according to the target population, which determines the source of the data to use, to build the questionnaire. In this study, we used the most recent national food consumption survey (INCA2), which dates from 2006. Some items should be added in the future to represent more closely today’s consumption patterns. The 94 items were based on an aggregation of the individual foods declared as consumed by adults in the INCA2 survey. The choice of the food aggregation was done by expertise, but another way to aggregate the foods could lead to different food and nutrient intake estimates. Another limitation of this study is the different time frame over which food intake was assessed by the reference method (dietary record on seven consecutive days) and will be assessed by the FFQ (aimed at assessing food intakes for the previous month). FFQs are typically designed to measure long-term food intake, conversely to dietary records which measure short-term intake. But, it could be assumed that the dietary data collection on day 7 is representative of the habitual consumption pattern of the individual. Moreover, the data used for the construction and validation were from the same study. It would be necessary to apply this approach with another open-ended food consumption survey. Despite the fact that the French FFQ showed an acceptable validity against the dietary survey used as a reference, the error estimated in this study did not represent the overall error when the FFQ will be used in practice with individuals (31). Indeed, self-report of food intakes could be biased by social desirability, which usually tend to overestimate intakes of foods considered “healthy” and underestimate less “healthy” foods (32–35). Validation of the individual’s perception of this questionnaire should be investigated further in the future.

The novelty of this study was to explore the impact on food and nutrient intakes estimates of the inherent structure of a FFQ. Usually, validation is done by comparing food and nutrient intakes estimated from the FFQ and a reference method (i.e., 24 h-recall or dietary record), completed by the same respondent under the same period. The reference method is supposed to quantify the same measure (i.e., food intakes) and should be independent of the FFQ, to avoid an interdependence of errors (36). However, measurement errors in validity measurement can also be attributable to the reference method. A better option would be to validate the nutrient intakes questionnaire estimates against biomarkers, but it is often too expensive and difficult to implement. This new “structural validation” method provides a first insight into validity of a FFQ by decomposing the measurement error according to its source (the use of an aggregated food database or the estimation of food quantity), independently of respondent induced bias and possible correlation errors. To date, the few FFQs which have been developed in French are either not recent (37, 38), longer than we needed (6, 29, 38, 39), or designed for a specific population or food group (40–42) and often not freely accessible. The FFQ presented in this study will be a useful tool to assess the usual food and nutrient intakes of French individuals. In a near future, a web-based version of this questionnaire will be used for French adults. Such tool will enable to assess easily the habitual diet of individuals to be used in ongoing studies focusing on monitoring usual behavior. Web-based versions have shown similar accuracy when compared to printed version (5, 43). They were also recognized to facilitate the collection of data (immediate storage), to reduce errors *via* automatic control, and are less burdensome for respondent than paper versions (44, 45). Moreover, the questionnaire can be personalized (adding complementary questions or removing one) according to the previous responses of the respondents.

## Conclusion

The “structural validation” presented in this study demonstrated that, without taking into account the respondent induced bias, the FFQ of 94 items designed for French adults provides reliable estimates of food and nutrient intakes for average consumers but with an overall trend to underestimated food and nutrient intakes. Further work would be required to validate the reproducibility and understanding of the questionnaire by respondents.

## Author Contributions

RG contributed to the design of the study, performed the statistical analysis, interpreted the results, wrote the manuscript, and was responsible for the final content of the manuscript; MM and FV contributed to the design of the study, help to interpret the results, and to produce the final draft of the manuscript; ND helped to produce the final draft of the manuscript; and all authors: read and approved the final version of the manuscript.

## Conflict of Interest Statement

The authors declare that the research was conducted in the absence of any commercial or financial relationships that could be construed as a potential conflict of interest. The reviewer LD and handling editor declared their shared affiliation.

## Abbreviations

FFQ, Food frequency questionnaire; INCA2, French Individual and National Dietary Survey 2; ANSES, French Agency for Food, Environmental and Occupational Health Safety; CNIL, French authority of data protection (“Commission Nationale Informatique et Libertés”); CNIS, French national council for statistical information (“Conseil National de l’Information Statistique”); EPA, eicosapentaenoic acid; DHA, docosahexaenoic acid.
