# Supplementary material for: Structural Validation of a French Food Frequency Questionnaire of 94 Items
Source: Front Nutr. 2017 Dec 20;4:62. doi: 10.3389/fnut.2017.00062 (PMC5742348; doi:10.3389/fnut.2017.00062)
Supplement: Supplementary file 1 [file Table_1.DOCX]

Supplementary Material

**Structural validation of a French food frequencyquestionnaire of 94 items**

**Rozenn Gazan, Florent Vieux, Nicole Darmon*, Matthieu Maillot**

*** Correspondence:** Corresponding Author: [nicole.darmon@inra.fr](mailto:nicole.darmon@inra.fr)

1. Supplementary Tables

**Table S1.** Food group and subgroups categorization

| **Food groups** | **Food sub-groups** | **Total number of items** | **Number of items with portion sizes requested** |
| --- | --- | --- | --- |
| **Fruits and vegetables** | Vegetables | 2 | 2 |
|  | Fresh and processed fruits | 3 | 0 |
|  | Nuts and oilseeds | 2 | 0 |
| **Starches** | Breads | 3 | 3 |
|  | Starches and legumes | 3 | 3 |
|  | Potatoes | 2 | 2 |
|  | Cereals for breakfast | 2 | 0 |
| **Meat/Fish/Eggs and substitutes** | Eggs | 1 | 1 |
|  | Fish | 3 | 0 |
|  | Meats | 4 | 0 |
|  | Deli meats | 2 | 1 |
|  | Offals | 1 | 0 |
|  | Protein substitutes as tofu | 1 | 0 |
| **Mixed dishes and sandwiches** | Soups | 2 | 0 |
|  | Mixed dishes | 7 | 0 |
|  | Sandwiches, snacks and salt pastries | 6 | 3 |
| **Dairy products and substitutes** | Milk | 3 | 3 |
|  | Yoghurt | 4 | 0 |
|  | Cheese | 2 | 2 |
|  | Vegetal substitutes | 1 | 0 |
| **Sweet products** | Ice creams and dairy desserts | 1 | 0 |
|  | Cakes, tarts, pastries and biscuits | 3 | 1 |
|  | Sweets | 4 | 2 |
| **Water and other beverages** | Water | 2 | 0 |
|  | Hot drinks | 3 | 3 |
|  | Light drink | 1 | 1 |
|  | Sweet drinks | 1 | 1 |
|  | Fruit juices | 1 | 1 |
|  | Alcohol | 3 | 3 |
| **Fats and condiments** | Animal fats | 6 | 5 |
|  | Vegetal fats | 8 | 8 |
|  | Hot sauces | 2 | 0 |
|  | Cold sauces | 4 | 4 |
|  | Salt | 1 | 1 |
