# Supplementary material for: Structural Validation of a French Food Frequency Questionnaire of 94 Items
Source: Front Nutr. 2017 Dec 20;4:62. doi: 10.3389/fnut.2017.00062 (PMC5742348; doi:10.3389/fnut.2017.00062)
Supplement: Supplementary file 2 [file Table_2.DOCX]

Supplementary Material

**Structural validation of a French food frequencyquestionnaire of 94 items**

**Rozenn Gazan, Florent Vieux, Nicole Darmon*, Matthieu Maillot**

*** Correspondence:** Corresponding Author: [nicole.darmon@inra.fr](mailto:nicole.darmon@inra.fr)

1. Supplementary Tables

**Table S2** Spearman correlation coefficients and cross-classification into quartiles between ‘REF_FOOD’ and “FFQ_FOOD’ food group and subgroup intakes, among consumers only

|  |  | Spearman correlation | | Cross-classification into quartiles | | | |
| --- | --- | --- | --- | --- | --- | --- | --- |
| **Food groups** | *N^a^* | *Raw* | *Partial^b^* | *Exact agreement (%)* | *Exact agreement plus adjacent (%)* | *Disagreement (%)* | *Extrem disagreement (%)* |
| **Fruits and vegetables** | 1850 | 0.94 | 0.93 | 73.0 | 99.2 | 0.8 | 0.1 |
| Vegetables | 1839 | 0.94 | 0.94 | 76.4 | 99.3 | 0.7 | 0.0 |
| Fresh and processed fruits | 1677 | 0.94 | 0.94 | 68.1 | 98.3 | 1.7 | 0.0 |
| Nuts and oilseeds | 502 | 0.99 | 0.99 | 54.8 | 87.9 | 11.0 | 1.2 |
| **Starches** | 1862 | 0.96 | 0.94 | 80.8 | 99.6 | 0.4 | 0.1 |
| Breads | 1829 | 0.98 | 0.97 | 87.3 | 99.6 | 0.4 | 0.0 |
| Starches and legumes | 1746 | 0.96 | 0.96 | 67.2 | 97.3 | 2.8 | 0.0 |
| Potatoes | 1681 | 0.97 | 0.97 | 77.9 | 99.8 | 0.2 | 0.0 |
| Cereals for breakfast | 318 | 1 | 1 | 55.0 | 95.9 | 4.1 | 0.0 |
| **Meat/Fish/Eggs and substitutes** | 1861 | 0.82 | 0.8 | 59.8 | 94.8 | 4.9 | 0.3 |
| Eggs | 1172 | 0.99 | 0.99 | 67.5 | 98.1 | 1.9 | 0.0 |
| Fish | 1482 | 0.88 | 0.88 | 52.2 | 93.6 | 6.1 | 0.3 |
| Meats | 1819 | 0.87 | 0.86 | 63.4 | 97.4 | 2.5 | 0.2 |
| Deli meats | 1689 | 0.89 | 0.88 | 63.2 | 96.2 | 3.4 | 0.4 |
| Offals | 422 | 0.99 | 0.99 | 37.7 | 77.0 | 18.7 | 4.3 |
| Protein substitutes as tofu | 17 | 1 | 1 | 94.1 | 94.1 | 5.9 | 0.0 |
| **Mixed dishes and sandwiches** | 1844 | 0.93 | 0.93 | 71.9 | 99.0 | 1.0 | 0.0 |
| Soups | 1003 | 0.99 | 0.99 | 66.2 | 97.8 | 2.2 | 0.0 |
| Mixed dishes | 1563 | 0.91 | 0.91 | 60.1 | 96.2 | 3.8 | 0.0 |
| Sandwiches. snacks and salt pastries | 1568 | 0.97 | 0.97 | 73.5 | 99.2 | 0.8 | 0.1 |
| **Dairy products and substitutes** | 1842 | 0.97 | 0.97 | 82.0 | 99.7 | 0.3 | 0.0 |
| Milk | 916 | 0.99 | 0.99 | 75.7 | 99.2 | 0.7 | 0.1 |
| Yoghurt | 1462 | 0.98 | 0.98 | 79.2 | 98.6 | 1.4 | 0.1 |
| Cheese | 1728 | 0.95 | 0.94 | 71.5 | 99.4 | 0.5 | 0.1 |
| Vegetal substitutes | 100 | 1 | 1 | 66.0 | 96.0 | 4.0 | 0.0 |
| **Sweet products** | 1825 | 0.9 | 0.88 | 66.7 | 97.6 | 2.4 | 0.1 |
| ice creams and dairy desserts | 1074 | 0.98 | 0.97 | 62.4 | 98.2 | 1.7 | 0.1 |
| Cakes. tarts and pastries | 1683 | 0.9 | 0.88 | 62.0 | 96.8 | 3.0 | 0.2 |
| Biscuits and sweets | 1684 | 0.92 | 0.92 | 70.8 | 97.3 | 2.2 | 0.5 |
| **Water and other beverages** | 1861 | 0.82 | 0.82 | 59.9 | 94.7 | 4.9 | 0.4 |
| Water | 1794 | 0.95 | 0.95 | 74.3 | 99.4 | 0.6 | 0.0 |
| Hot drinks | 1699 | 0.92 | 0.92 | 66.2 | 97.6 | 1.9 | 0.5 |
| Light drink | 218 | 1 | 1 | 93.1 | 99.5 | 0.5 | 0.0 |
| Sweet drinks | 640 | 1 | 1 | 88.8 | 98.4 | 1.6 | 0.0 |
| Fruit juices | 905 | 0.99 | 0.99 | 68.8 | 98.9 | 1.1 | 0.0 |
| **Fats and condiments** | 1858 | 0.93 | 0.92 | 75.7 | 98.9 | 1.1 | 0.1 |
| Animal fats | 1627 | 0.98 | 0.98 | 83.2 | 99.8 | 0.3 | 0.0 |
| Vegetal fats | 1703 | 0.98 | 0.98 | 79.7 | 99.9 | 0.1 | 0.0 |
| Hot sauces | 1106 | 0.93 | 0.93 | 52.4 | 87.8 | 10.9 | 1.4 |
| Cold sauces | 1327 | 0.99 | 0.99 | 84.4 | 99.9 | 0.1 | 0.0 |
| Salt | 438 | 1 | 1 | 85.2 | 96.8 | 2.3 | 0.9 |

***^a^*** *Number of consumers by food groups and subgroups*

***^b^*** *Adjustment for “REF_NUT” total energy intake*
