# Supplementary material for: Structural Validation of a French Food Frequency Questionnaire of 94 Items
Source: Front Nutr. 2017 Dec 20;4:62. doi: 10.3389/fnut.2017.00062 (PMC5742348; doi:10.3389/fnut.2017.00062)
Supplement: Supplementary file 3 [file Table_3.DOCX]

Supplementary Material

**Structural validation of a French food frequencyquestionnaire of 94 items**

**Rozenn Gazan, Florent Vieux, Nicole Darmon*, Matthieu Maillot**

*** Correspondence:** Corresponding Author: [nicole.darmon@inra.fr](mailto:nicole.darmon@inra.fr)

1. Supplementary Tables

**Table S3.** Spearman correlation coefficients and cross-classification into quartiles between ‘REF_NUT and ‘FFQ_NUT’ daily energy and nutrient intakes

|  | **Spearman Correlation** | | **Cross-classification into quartiles** | | | |
| --- | --- | --- | --- | --- | --- | --- |
|  | *Raw* | *Partial^a^* | *Exact agreement (%)* | *Exact agreement plus adjacent (%)* | *Disagreement (%)* | *Extrem disagreement (%)* |
| Energy (kcal/d) | 0.87 | . | 63.0 | 97.0 | 3.0 | 0.1 |
| Energy (kJ/d) | 0.87 | . | 63.0 | 97.0 | 3.0 | 0.1 |
| Proteins (% energy) | 0.82 | 0.81 | 58.6 | 94.2 | 5.5 | 0.3 |
| Carbohydrates (% energy) | 0.87 | 0.87 | 63.4 | 97.1 | 2.9 | 0.1 |
| Total sugar (% energy) | 0.90 | 0.90 | 65.7 | 97.9 | 2.0 | 0.1 |
| Free sugars (% energy) | 0.84 | 0.84 | 64.2 | 95.9 | 3.6 | 0.5 |
| Total fat (% energy) | 0.86 | 0.86 | 62.2 | 95.9 | 4.0 | 0.1 |
| Saturated fat (% energy) | 0.86 | 0.86 | 61.5 | 96.1 | 3.7 | 0.2 |
| Mono insaturated fat (% energy) | 0.85 | 0.86 | 61.0 | 96.2 | 3.7 | 0.2 |
| Poly insaturated fat (% energy) | 0.88 | 0.88 | 64.5 | 97.0 | 2.7 | 0.3 |
| Fiber (g/d) | 0.87 | 0.77 | 63.3 | 96.9 | 3.0 | 0.1 |
| Water (g/d) | 0.89 | 0.88 | 67.9 | 97.6 | 2.3 | 0.1 |
| EPA (g/d) | 0.84 | 0.83 | 82.4 | 82.4 | 17.6 | 0.0 |
| DHA(g/d) | 0.82 | 0.81 | 68.8 | 82.3 | 16.1 | 1.7 |
| α-Linolenic acid (g/d) | 0.77 | 0.66 | 53.1 | 92.1 | 7.3 | 0.6 |
| Linoleic acid (g/d) | 0.89 | 0.83 | 65.0 | 97.6 | 2.2 | 0.2 |
| Sodium (g/d) | 0.87 | 0.74 | 62.3 | 96.9 | 3.1 | 0.1 |
| Potassium (mg/d) | 0.83 | 0.70 | 60.2 | 95.2 | 4.5 | 0.3 |
| Magnesium (mg/d) | 0.79 | 0.65 | 56.7 | 93.3 | 6.2 | 0.5 |
| Calcium (mg/d) | 0.84 | 0.77 | 60.4 | 95.8 | 3.8 | 0.4 |
| Copper (mg/d) | 0.72 | 0.56 | 52.1 | 89.5 | 9.1 | 1.3 |
| Iron (mg/d) | 0.78 | 0.59 | 53.3 | 92.7 | 6.7 | 0.6 |
| Phosphorus (mg/d) | 0.82 | 0.61 | 58.4 | 94.5 | 5.4 | 0.1 |
| Iodine (g/d) | 0.74 | 0.62 | 52.1 | 91.5 | 7.7 | 0.8 |
| Zinc (g/d) | 0.80 | 0.65 | 57.7 | 93.0 | 6.4 | 0.5 |
| Selenium (g/d) | 0.71 | 0.63 | 52.7 | 89.4 | 9.2 | 1.4 |
| Vitamin A(ER/d) | 0.66 | 0.58 | 49.2 | 87.0 | 10.9 | 2.1 |
| Vitamin C(mg/d) | 0.86 | 0.85 | 63.2 | 96.7 | 3.1 | 0.2 |
| Vitamin D(µg/d) | 0.72 | 0.66 | 50.7 | 90.7 | 8.2 | 1.1 |
| Vitamin E(mg/d) | 0.89 | 0.86 | 67.9 | 97.3 | 2.7 | 0.0 |
| Thiamin (mg/d) | 0.80 | 0.69 | 56.2 | 93.5 | 5.7 | 0.9 |
| Riboflavin (mg/d) | 0.81 | 0.73 | 57.6 | 92.4 | 6.8 | 0.8 |
| Niacin (mg/d) | 0.75 | 0.64 | 52.4 | 92.3 | 6.7 | 1.0 |
| Panthotenic acid (mg/d) | 0.80 | 0.66 | 57.3 | 93.6 | 6.0 | 0.4 |
| Vitamin B-6 (mg/d) | 0.80 | 0.69 | 56.4 | 93.7 | 6.1 | 0.2 |
| Folate (µg/d) | 0.81 | 0.72 | 56.8 | 94.5 | 5.1 | 0.4 |
| Vitamin B-12 (µg/d) | 0.72 | 0.67 | 52.8 | 89.1 | 9.6 | 1.4 |

***^a^*** *Adjustment for “REF_NUT” total energy intake*
