# Supplementary figures and images for: Structural Validation of a French Food Frequency Questionnaire of 94 Items
Source: Front Nutr. 2017 Dec 20;4:62. doi: 10.3389/fnut.2017.00062 (PMC5742348; doi:10.3389/fnut.2017.00062)

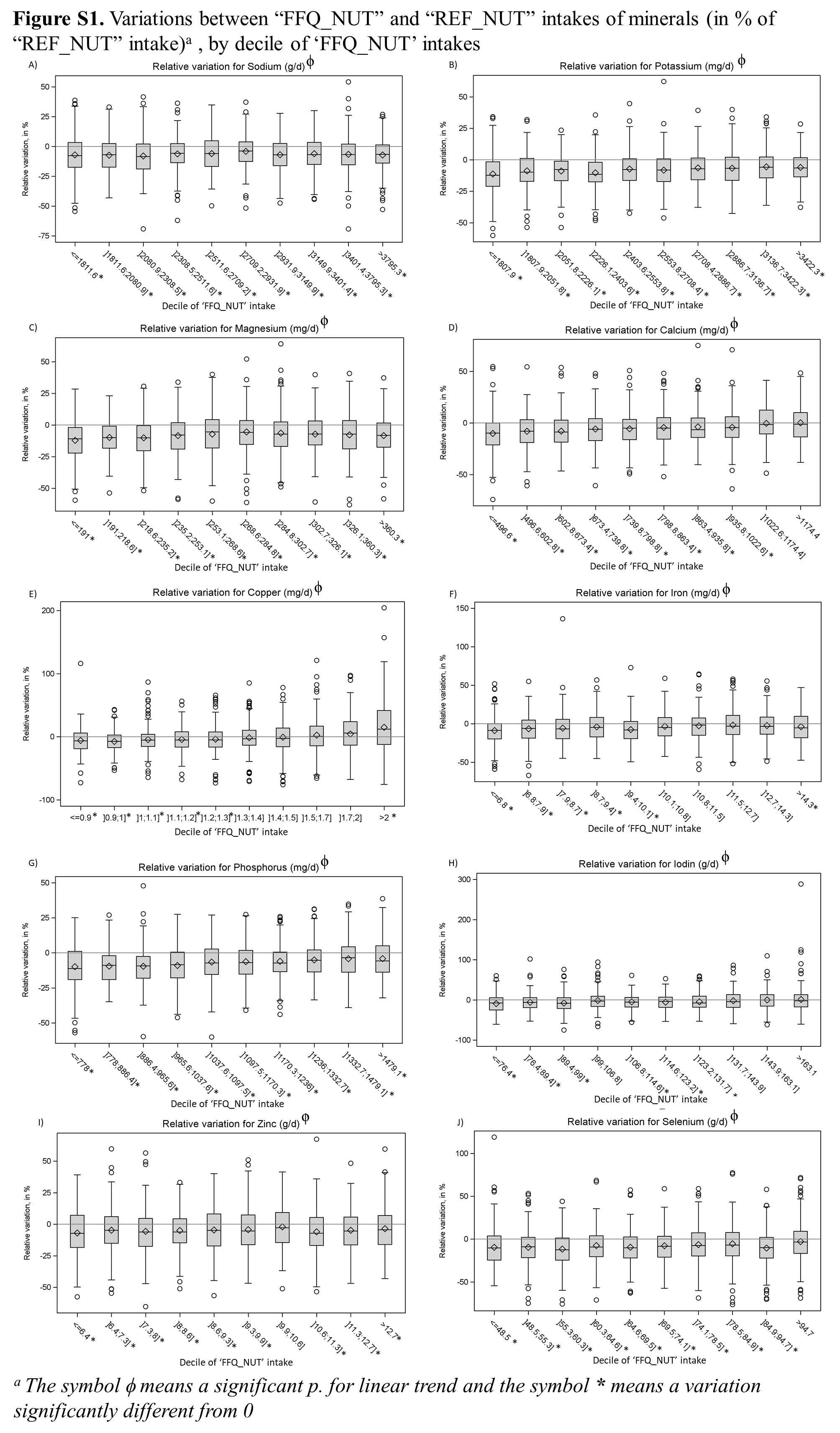

Supplement: Supplementary file 4 [file Image_1.PNG]

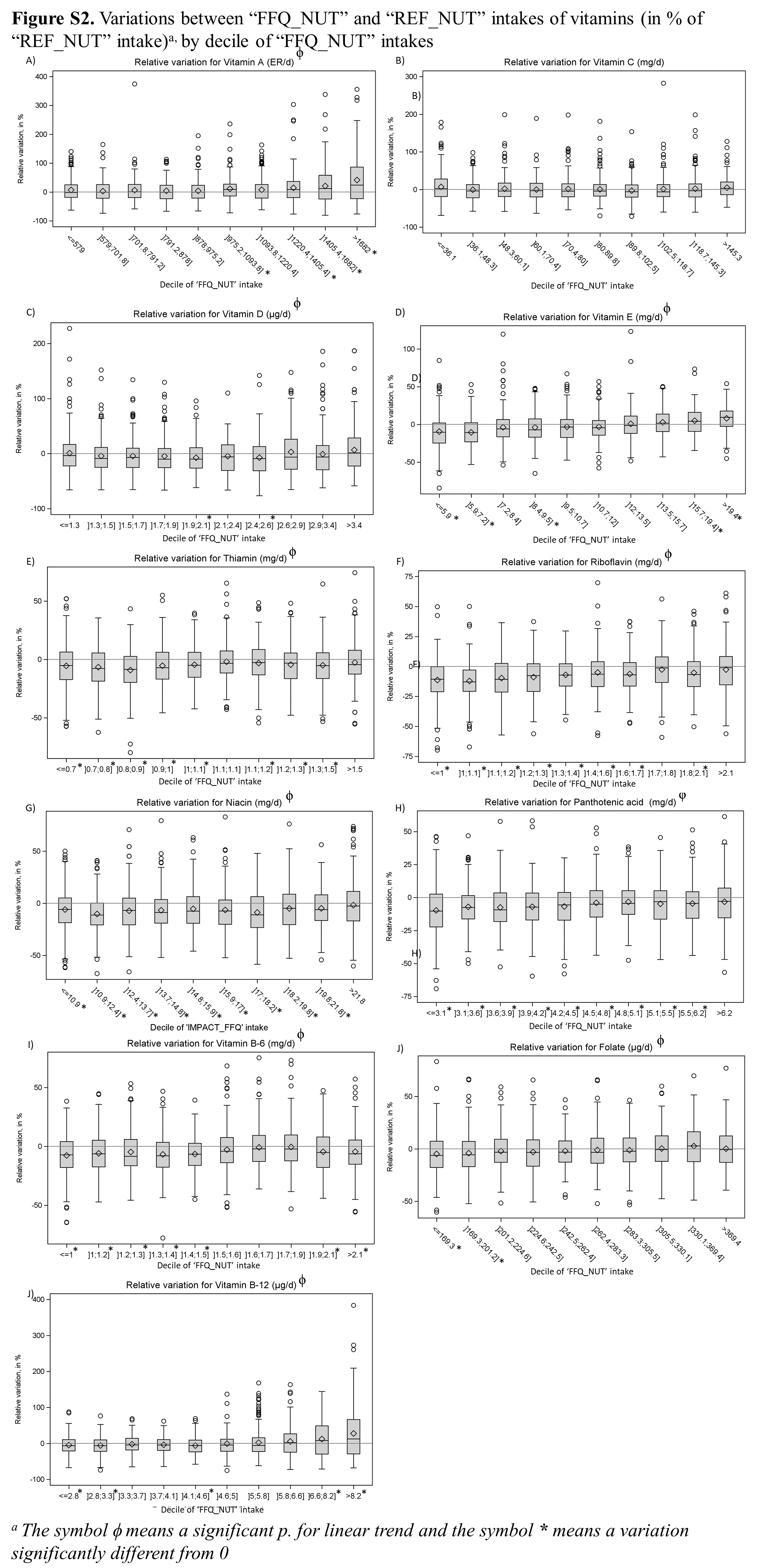

Supplement: Supplementary file 5 [file Image_2.PNG]
